# Cyberbullying Based on Social Stigmas and Social, Emotional and Moral Competencies

**DOI:** 10.3390/bs15050646

**Published:** 2025-05-09

**Authors:** Antonio J. Rodríguez-Hidalgo, Victoria S. Camargo, Almudena Hurtado-Mellado

**Affiliations:** Department of Psychology, University of Cordoba, 14071 Cordoba, Spain; ajrodriguez@uco.es (A.J.R.-H.); m72sacav@uco.es (V.S.C.)

**Keywords:** cyberbullying, social stigma-based cyberbullying, moral disengagement, moral emotions, socioemotional competences

## Abstract

Cyberbullying is a violent phenomenon that threatens health and development in adolescence. Some studies suggest that minority groups or those who deviate from socially desirable characteristics are at a greater risk of cyberbullying. However, there have been few studies on social stigma-based cyberbullying (SSB). This study aims to carry out the following: to know the prevalence of roles of involvement in cyberbullying and SSB cyberbullying; to understand the possible relationships between SSB cybervictimization and SSB cyberaggression and the different dimensions of moral disengagement, moral emotions and social and emotional competencies; and to know the possible statistical associations between roles and the variables described above. A total of 601 secondary school students took part in this study, aged 12–19 (M = 14.22, SD = 1.355). A self-report battery of scales was employed to measure the constructs under investigation, and various statistical analyses were conducted. The results show that 22.29% are recognized as cybervictims, 7.82% as cyberaggressors and 35.11% as cybervictims/cyberaggressors in general cyberbullying. In SSB cyberbullying, the percentages were 20.30%, 3% and 8.32%, respectively. Moreover, it was revealed that SSB cyberaggression was related to moral disengagement and SSB cybervictimization was related to moral emotions. Social competence and emotional competence were positively related to social stigma-based cybervictimization. The results are discussed and new lines of research and interventions focused on social competences and moral emotions are proposed.

## 1. Introduction

The use of Information and Communication Technologies (ICTs) has revolutionized the way people communicate and interact with each other (Kowalski et al., 2019). Today, people aged 12 to 25 are part of the so-called “digital generation”. This group that has grown in an environment where there is a daily use of video calls, instant messaging and social networks (Gómez-Urrutia & Jiménez Figueroa, 2022). In Spain, the latest survey on the equipment and use of information and communication technologies in households carried out by the National Institute of Statistics (INE, 2024) shows that the age group with the highest percentage of Internet use is 16–24 year olds with a value of 99.8% (INE, 2024). The same report shows that 96% of children aged 10 to 15 use the Internet and 69.6% have a smartphone, with a higher prevalence of this behavior in girls than boys: 96.5% and 70.9%, respectively. When comparing these findings to data from the same survey two years earlier (INE, 2022), it is evident that Internet usage has increased.

The world of new technologies and access to them provides a framework that benefits and offers new opportunities for academic, social and personal development. However, this new scenario also carries important risks, such as cyberbullying, which begins to emerge in early childhood and adolescence and influences the normative development of individuals (Kowalski et al., 2019). It is also important to highlight that not all individuals are affected in the same way. Those who differ from the group for any reason are at a heightened risk of becoming involved in these violent dynamics (Calmaestra et al., 2020). This makes it essential to study this phenomenon in greater depth, taking these considerations into account.

### 1.1. Cyberbullying

Cyberbullying is harassment that occurs through electronic devices, social media and online games, where the attacks are intentional and target victims who have difficulty defending themselves (Cuadrado et al., 2019; Marinoni et al., 2024b; Smith et al., 2008). This phenomenon, which occurs in an online environment, involves two main processes: cyberaggression and cybervictimization. It is characterized by dynamic social interactions in which individuals assume different roles, such as the cyberaggressor, cybervictim, or both: cyberaggressor/cybervictim (Del Rey et al., 2015; Rodríguez-Hidalgo et al., 2018). Cyberbullying is an aggressive and harmful phenomenon related to antisocial behaviors present in society, which affects the academic, personal and social development of young people (Kowalski et al., 2019).

Cyberbullying involves behaviors present in traditional bullying, such as the display of shameful images, verbal attacks and exclusion from the group, but in this case, through electronic devices. Cyberbullying can also involve behaviors such as impersonating another person or hacking personal accounts to obtain private information among other behaviors (Del Rey et al., 2015). Both phenomena share key characteristics in terms of power imbalance, intentionality and repetition. But this last element, in the case of cyberbullying, is a point of controversy since it is not necessary for aggressive behavior to be repeated for the harmful effects to occur (Ortega-Ruiz, 2018). Additionally, the power imbalance in cyberbullying differs slightly from traditional bullying, as the cyberaggressor does not need to be physically larger or stronger; instead, they only need the knowledge and skills to use digital devices in cruel and harmful ways against the cybervictim (Zych & Llorent, 2023). Moreover, the anonymity afforded by fake profiles, along with the increased amount of time spent online, places young people at greater risk of becoming involved in this phenomenon (Marinoni et al., 2024a).

A report in which Save the Children investigated the cyberbullying of young people aged 12 to 16 tried to draw the profile of victims and aggressors of this phenomenon, finding that 6.9% have suffered cyberbullying, with a higher percentage of girls being the object of it. Regarding cyberaggression, the prevalence in this report reveals that 5.4% admit to having bullied someone, highlighting boys in this role more than girls (Calmaestra et al., 2016).

### 1.2. Cyberbullying Based on Social Stigmas

In recent decades, cyberbullying has been a well-studied phenomenon. However, in most cases, aspects linked to human diversity and social group dynamics have not been considered, such as feeling part of a group that is identified as different by others and prejudices, among others. In this context, it is important to take into account social stigma, understood as the phenomenon by which a group is labeled—typically through the assignment of a name—creating a linguistic separation from the rest of society, becoming negatively stereotyped, and being subjected to a power imbalance in relation to other members of that society (Andersen et al., 2022). One of the basic elements for a cyberbullying dynamic to be generated is the power imbalance between aggressors and victims. In studies on cyberbullying carried out to date, the power imbalance in cyberbullying—which has been studied little—has its origin in the perceived or attributed differences between those involved, based on social stigmas based on human diversity traits linked to gender identity and/or sexual orientation, ethnic–cultural identity, having special educational needs and/or disability and appearance or physical capacity, among others. In this regard, according to Social Dominance Theory, those who engage in peer harassment may be seen as reproducing or, to some extent, reaffirming preexisting social hierarchies in order to maintain or enhance their advantaged position relative to others (Goodboy et al., 2016; Vaillancourt et al., 2003).

The power imbalance in social stigma-based cyberbullying may be rooted in discrimination based on prejudice that some groups of people suffer from because of a particular characteristic—such as belonging to an ethnic–cultural minority group or sexual orientation—which places members of minority groups at greater risk of bullying (Calmaestra et al., 2020; Rodríguez-Hidalgo et al., 2018; Wright & Wachs, 2021). Some studies have shown that ethnic–cultural and sexual minority groups are at a high risk of being involved in cyberbullying (Zych & Llorent, 2023). Graham (2021) considers bullying based on an ethnic–cultural factor as a subtype of social stigma-based bullying; other reasons such as sexual identity may be involved. Therefore, someone may be bullying at the same time due to two different identity backgrounds.

In Spain, it has been observed that adolescents from the majority ethnic–cultural group, payos—a term used in communities in Spain to refer to persons of the majority cultural group as opposed to belonging to the Gypsy or Romani identity—are least involved in cyberbullying and that Gypsy or Romani adolescents stand out in the role of cyberaggressor and cybervictim/cyberaggressor (Calmaestra et al., 2020). In addition, regarding a group of young immigrants, it was observed that first-generation people were more often involved as cybervictims/cyberaggressors and second-generation people as cybervictims. In the same line, Rodríguez-Hidalgo et al. (2018) indicate that their study did not find significant differences in cyberaggression between different ethnic–cultural groups, but Gypsy or Romanian teenagers were more cybervictimized than most teens born in Spain.

Regarding cyberbullying related to sexual orientation, in the study by Elipe et al. (2017), they observed that most subjects who self-identified as heterosexual recognized themselves as a cybervictim (12%), while the minority who self-identified as non-heterosexual were recognized as cybervictims in 21% of cases. Another study conducted in the Basque Country by Garaigordobil and Larrain (2020) showed that the percentage of cybervictims was significantly higher in minorities due to sexual orientation, suffering from more aggressive cyberbullying than the rest of their heterosexual peers. Also, in the study by Llorent et al. (2016), it was found that the score for both victimization and aggression was higher among minorities due to sexual orientation.

The Fundación ANAR and Fundación Mutua Madrileña (2022) carried out a study that highlights reasons for perpetrating bullying in schools, revealing that 56.5% of cases of victimization were due to differences in physical appearance, which can be related to ethnic–cultural or skills and abilities. In the study by De Oliveira et al. (2015), almost one fifth of the students pointed out that the appearance of the body and the appearance of the face were identified as causal reasons for the bullying phenomenon. In the same line, it observed that physical appearance and body image are one of the main reasons for becoming a victim of bullying (Rech et al., 2013). Body image is significantly associated with the victim role, with being thin or very thin constituting the main reasons (Reulbach et al., 2013). However, in a review of the scientific literature, references have not been found regarding cyberbullying based on social stigmas linked to physical appearance.

Despite these findings, of the majority of studies on cyberbullying, only a few consider comparing groups based on some identity traits; and the study of cyberbullying specifically based on social stigma remains a challenge and an emerging line of research.

### 1.3. Cyberbullying and Moral Disengagement

There is a wide variety of research focusing on the risk and protective factors of cyberbullying. However, most of these are studied independently and different elements need to be included to understand the relationship between them. Hostile contexts can affect people’s moral judgment, producing a reciprocal and bidirectional relationship between morality and aggression. In the context of bullying and cyberbullying—where immoral behavior is being carried out—there may be an increase in moral disconnection, understanding this process as a series of cognitive mechanisms that some people use to break away from their moral standards and justify immoral actions (Bandura, 2002). In addition, eight moral disengagement mechanisms have been described and grouped into four broad categories: cognitive restructuring, minimizing responsibility, distorting the consequences and dehumanizing the victim. These mechanisms of moral disengagement, which allow the aggressor to self-regulate their cognitive processes in order to avoid negative consequences when harming another person, are closely associated with both face-to-face bullying and cyberbullying, mainly with the role of aggressor (Romera et al., 2019a, 2021).

In a meta-analysis that included 27 studies from different countries related to moral disengagement and aggressiveness in children and adolescents, it was revealed that low morality was directly related to aggressiveness and could be related to cyberbullying (Gini et al., 2014). It has been observed that the mechanisms of moral disengagement are related to a greater predisposition to commit traditional bullying (Zych & Llorent, 2019). In the same line, it has been shown that the mechanisms of moral disengagement are associated with cyberbullying, with cognitive restructuring being the most associated mechanism and specifically promoting aggression in younger students (Romera et al., 2021).

A recent study by Domínguez-Vergara et al. (2023) revealed data that go in the same direction, showing a positive association between moral disconnection and cyberbullying, in which cybervictims and cyberaggressors use cognitive mechanisms of moral disengagement, thus avoiding feeling guilty.

### 1.4. Cyberbullying and Moral Emotions

Moral emotions are defined as adaptive emotions that appear after moral transgressions and promote moral actions (Ortega-Ruiz et al., 2002). They are conscious emotions generated by a person’s self-assessment and are important for the development of moral actions because they motivate prosocial behavior and inhibit immoral and antisocial behavior. In addition, it is important to consider moral emotions as part of moral reasoning as it promotes the ability to appreciate the emotional consequences that our actions have on others (Romera et al., 2019a). Cyberbullying, in this sense, is usually associated with a low experience of moral emotions (Oriol Granado, 2023).

Positive moral emotions are generally related to the maintenance of established actions, while negative emotions promote behavior change (Baumeister et al., 2007). Moral emotions have been observed to help prevent negative behaviors because they anticipate negative outcomes (Arsenio, 2014). A study with adolescents aged 12–19 found that the abusers reported low levels of moral emotions (Perren & Gutzwiller-Helfenfinger, 2012). On the other hand, Romera et al. (2019b), through hypothetical situations, found that those who were involved as victims of bullying associated the victim with emotions of indifference, while those not involved attributed emotions of embarrassment; these results suggest that victims of traditional bullying actively hide their emotional response.

### 1.5. Cyberbullying and Emotional and Social Competencies

Studies focus on social and emotional competencies in relation to bullying and cyberbullying as a protective factor. Social and emotional competencies are defined as the ability to use knowledge and abilities in a positive way to perform prosocial behaviors in different social contexts (Llorent et al., 2021).

According to Eden et al. (2016), students who have high social and emotional skills are less likely to engage in bullying and cyberbullying. It has also been observed that cybervictims score high in social competence and victimized cyberaggressors score high in popularity targets and low in social acceptance, concluding that the way adolescents manage their social and emotional competencies may be an explanation for this phenomenon (Romera et al., 2016). In addition, an emotional competence development program conducted in adolescents between 15 and 17 years of age had a positive impact on cyberbullying scores (Martínez-Vilchis et al., 2018). Thus, cyberbullying seems to have an inverse relationship with emotional competencies, with adolescents with a lower level of these being more likely to be involved in this violent phenomenon (Solsol-Urquía, 2022).

### 1.6. The Present Study

There are different research studies focusing on moral disengagement, social and emotional competencies and moral emotions in relation to cyberbullying. However, little is known about the possible relationship between these topics and cyberbullying based on social stigmas.

The study of these variables would make it possible to know if there are relationships between them and which ones are linked to cyberbullying based on social stigmas (cybervictimization and cyberaggression).

On the other hand, although several studies find that minority groups are at a higher risk of becoming involved in cyberbullying, there is still little awareness about social stigma-based cyberbullying, so tools to measure this phenomenon need to be generated, adapted and validated. The present study used the European Cyberbullying Intervention Project Questionnaire (ECIPQ, Ortega-Ruiz et al., 2016), and then a registration question was introduced to discriminate cases of cyberbullying based on social stigmas that were allowed to be registered on the grounds of differences in skin color, religion, culture, place of origin, Specific Educational Needs (SEN), sexual orientation, gender, physical and/or physical appearance and physical abilities or skills.

The objectives of this study were as follows: (a) to know the prevalence of roles of involvement—cyberaggressor, cybervictim and cyberaggressor/cybervictim—in general cyberbullying and in cyberbullying based on social stigmas, as well as the gender-related involvement; (b) to understand the potential connections between social stigma-based cyberaggression and social stigma-based cybervictimization and the different dimensions of moral disengagement, moral emotions, and social and emotional competencies; and (c) to know the possible associations between cyberaggression based on social stigmas, cybervictimization based on social stigmas and the variables described above.

Based on a review of the literature, the following hypotheses were put forward:

1. Involvement in the different roles—cyberaggressor, cybervictim and cyberaggressor/cybervictim—will be observed in cyberbullying based on social stigmas as it occurs in the phenomenon of general cyberbullying (Andrade et al., 2021; Calmaestra et al., 2016, 2020).
2. Boys will stand out over girls in their involvement in the roles of cyberaggressor both in general cyberbullying and in social stigma-based cyberbullying (Andrade et al., 2021; Baldry et al., 2015; Calmaestra et al., 2020).
3. Social stigma-based cyberaggression will be positively related to cybervictimization and moral disengagement (Gini et al., 2014; Rodríguez-Hidalgo et al., 2018; Romera et al., 2021; Zych & Llorent, 2019).
4. Cybervictimization based on social stigmas will be positively related to cyberaggression and moral emotions (Domínguez-Vergara et al., 2023; Oriol Granado, 2023; Romera et al., 2019a).
5. Social and emotional competencies will be positive related to social stigma-based cybervictimization (Solsol-Urquía, 2022; Zych & Llorent, 2023).

## 2. Materials and Methods

### 2.1. Study Design

This is a retrospective ex post facto transverse study (Montero & León, 2007) where quantitative information is recorded. The sample was selected through an intentional non-probabilistic sampling (Ruiz-Olabuénaga, 2012).

### 2.2. Participants

Participants were 601 adolescents enrolled in 3 public secondary schools in a town in Seville: 583 students in compulsory secondary education (97%) and 18 students in the intermediate cycle (3%). All participants studied in schools with similar characteristics in terms of ethnic, cultural and socio-economic background. The participants’ age was between 12 and 19 (M = 14.22, SD = 1.355), with 45.1% women, 53.6% men and 0.8% gender non-binary (0.5% did not reveal their gender).

### 2.3. Procedure

The different high schools were contacted by e-mail requesting an in-person meeting to explain the different objectives of the study, which was held with the counselors from the different centers. The management of the centers approved participation in the study and confidentiality documents and authorizations were signed. In addition, written consent was obtained from both participants and their parents or legal guardians.

The data were collected using paper-based questionnaires, which were distributed class by class, except in one of the schools, where administration took place in the auditorium. In both settings, participants completed the questionnaires individually in an environment designed to facilitate focused responses. Researchers were present at all times to administer the questionnaires and to address any questions or concerns that might arise. Participation was voluntary and anonymous, as students were informed about the purpose of the study and could object to answering the different scales at any time. The questionnaires were administered during the tutoring hours for approximately 40 min. The collection took place during the month of March 2023. The instruments used were in accordance with the principles of the Declaration of Helsinki.

### 2.4. Instruments

The measurement instrument used was a self-report battery consisting of a set of questionnaires. The first part of the instrument was aimed at collecting socio-demographic data such as gender, age, nationality, sexual orientation, educational needs, disability and religion, among others. The second part of the instrument was composed of the following questionnaires:

*Cyberbullying:* To evaluate involvement in cyberbullying, the Spanish version of the European Cyberbullying Intervention Project Questionnaire (ECIP-Q) by Del Rey et al. (2015) validated by Ortega-Ruiz et al. (2016) in Spain was used.

This questionnaire includes 22 items that are answered with a five-point Likert scale ranging from 0 (never) to 4 (yes, more than once a week) referring to a time interval of the last two months (α = 0.887). In addition, the items are divided into two dimensions: 11 items focused on cybervictimization (α = 0.845; for example, “Someone has said bad words or insulted me online”) and 11 items focused on cyberaggression (α = 0.858; for example, “I have said profanity to someone using Internet messages or SMS”).

To measure cyberbullying based on social stigmas, a list of 8 dichotomous variables was added after each dimension, providing information on different motives based on social stigmas for which participants have been cybervictims and/or cyberaggressors as marked in the ECIP-Q (e.g., “differences in skin colour, culture, religion or coming from elsewhere”).

*Moral Disengagement:* Moral disengagement was analyzed through the version validated in Spanish by Romera et al. (2023) of the Moral Disengagement Scale (MDS; Caprara et al., 1995), using the version for adolescents with 24 items called Moral Disengagement Scale-24 (MDS-24), with a reliability level of α = 0.894. These items have 5 Likert-type response options from 1 (strongly disagree) to 5 (totally agree) that measure the four dimensions of moral disengagement; cognitive restructuring (α = 0.786; for example, “It’s alright to beat someone who bad mouths your family”), minimizing responsibility (α = 0.773; for example, “Kids cannot be blamed for misbehaving if their friends pressured them to do it”), distorting the consequences (α = 0.608); for example, “It is okay to tell small lies because they don’t really do any harm”) and dehumanizing the victim (α = 0.764; for example, “Some people deserve to be treated like animals (in a cruel way)”).

*Moral emotions*: The Moral Emotions scale was used, which was designed and validated in young Spaniards between 9 and 19 years old by Álamo et al. (2019). This instrument is composed of 5 items, which are answered on a Likert scale from 1 (totally disagree) to 5 (totally disagree) (α = 0.833). Items relate to moral emotions that appear in the face of moral transgressions such as guilt, regret, pride and shame (for example, “I feel guilty after hurting a classmate”).

*Social and emotional competencies*: Social and emotional competencies were measured through the Social and Emotional Competences Questionnaire (SEC-Q) validated in students between 11 and 19 years old by Zych et al. (2018). This instrument consists of 16 items with 5 possibilities for Likert-type responses from 1 (totally disagree) to 5 (totally agree). In addition, this questionnaire (α = 0.902) is divided into four dimensions: self-awareness (α = 0.815, e.g., “I am aware of the thoughts that influence my emotions”), self-management and motivation (α = 0.813, e.g., “I know how to motivate myself”), social awareness and prosocial behavior (α = 0.831, e.g., “I pay attention to the needs of others”) and responsible decision making (α = 0.802, e.g., “I make decisions considering carefully possible consequences”).

### 2.5. Statistical Analysis

The analyses developed are quantitative and target-oriented, conducted to check whether the initial hypotheses are confirmed. The data collected were introduced and analyzed with the SPSS statistical package (version 28.0.1).

The variables of cyberaggression and cybervictimization based on social stigmas were generated, based on the students’ answers to filter questions regarding the reasons for both behaviors. In each situation, cases were selected where the student responded by pointing out at least one social stigma-based reason. Following this, frequency analyses were made based on bias and the gender of involved and non-involved participants. Normality tests were carried out to determine if the sample had a normal distribution. In addition, bivariate correlations of Spearman were analyzed among all variables of interest: social stigma-based cyberaggression, social stigma-based cybervictimization and dimensions of moral disengagement, such as cognitive restructuring, minimizing responsibility, distorting consequences, and dehumanizing the victim; moral emotions, including guilt, regret, shame, and pride; and social and emotional competences, such as self-awareness, self-management and motivation, social awareness and prosocial behavior, and responsible decision making. Finally, multiple linear regression was performed to identify the statistical associations of cyberaggression and cybervictimization based on social stigmas with the variables described above.

## 3. Results

### 3.1. Normality Tests

To assess the normality of the data, the Kolmogorov–Smirnov test was conducted, revealing significant deviations in both stigma-based cybervictimization and cyberaggression (*p* < 0.001). Descriptive statistics indicated a positive skewness (0.939) and negative kurtosis (−1.121) for stigma-based cybervictimization, as well as both positive skewness (2.449) and positive kurtosis (4.009) for stigma-based cyberaggression.

Given that the variables did not meet the assumption of normality—as evidenced by the results of the Kolmogorov–Smirnov test and the descriptive statistics (i.e., skewness and kurtosis values)—appropriate statistical procedures were employed to account for this deviation. Specifically, non-parametric tests were used in place of their parametric counterparts, as they do not require normally distributed data and are more robust under conditions of skewed distributions or heteroscedasticity. This approach ensures the validity and reliability of the results despite the distributional characteristics of the data.

### 3.2. Frequency of Involvement

An analysis of the frequency of involvement in cyberbullying as a general phenomenon and as a phenomenon based on social stigma was carried out (see Table 1). The data show that of the 601 students who answered the questionnaire, 22.29% (n = 134) have been involved as cybervictims, 7.82% (n = 47) as cyberaggressors and 35.11% (n = 211) in the role of cybervictim/cyberaggressor. The levels of involvement in stigma-based cyberbullying are notably lower than those observed in traditional cyberbullying; however, they follow a similar trend, with the highest levels corresponding to cyberaggressors—20.30% (n = 122)—followed by individuals occupying a mixed role of cybervictim/cyberaggressor—8.32% (n = 50)—and the lowest levels associated with cybervictims—3.00% (n = 18).

In addition, a frequency analysis of multiple-choice tables was performed to observe the reasons why students have been involved in cyberbullying based on social stigmas. These results reveal that the most common reasons for being involved as cybervictims are due to differences in body appearance with 80.23% (n = 138) and 21.52% due to differences in physical abilities or skills (n = 37). In the case of cyberaggression, the results are similar: 61.76% (n = 42) due to differences in appearance or body appearance and 22.05% (n = 15) due to differences in physical abilities or skills.

### 3.3. Relationship Between Cybervictimization and Cyberaggression Based on Social Stigmas and Moral Disconnection, Moral Emotions and Social and Emotional Competencies

Spearman bivariate correlation analysis was conducted to discern the relationship between social stigma-based cyberaggression and stigma-based cybervictimization variables in relation to the different dimensions of moral disengagement, moral emotions and social and emotional competences, as well as the interrelationships between them (see Table 2).

#### 3.3.1. Cybervictimization

Stigma-based cybervictimization is significantly correlated with stigma-based cyberaggression (*r* = 0.353, *p* < 0.01), which represents the strongest link.

In relation to moral disengagement, significant correlations were found with the dimensions of cognitive restructuring (*r* = 0.122, *p* < 0.01) and minimizing responsibility (*r* = 0.104, *p* < 0.01). No statistically significant relationships were observed with moral emotions. However, a significant negative correlation was found between the responsible decision-making dimension of social and emotional competencies and stigma-based cybervictimization (*r* = −0.085, *p* < 0.05).

#### 3.3.2. Cyberaggression

Considering cyberaggression based on social stigmas, we find significant correlations with cybervictimization based on stigmas, as indicated above, as well as with all dimensions of the moral disengagement scale, including cognitive restructuring (*r* = 0.190, *p* < 0.01), minimizing responsibility (*r* = 0.112, *p* < 0.01), distortion of consequences (*r* = 0.093, *p* < 0.05), and dehumanizing the victim (*r* = 0.132, *p* < 0.01).

However, no statistically significant correlations were found between stigma-based cyberaggression and moral emotions or social and emotional competencies.

### 3.4. Statistical Associations of Social Prejudice-Based Cybervictimization and Social Prejudice-Based Cyberaggression

For cybervictimization based on social stigma (see Table 3), the Durbin–Watson statistic showed a value of 1.874, and the collinearity analyses were optimal since the values of the variance inflation factor (VIF) were between 1.060 and 2.766 and the tolerance values were between 0.361 and 0.943. It was observed that 13.40% of the variance of cybervictimization based on social stigmas is explained by the variable of cyberaggression based on social stigmas (positive relation) and by the constructs of cognitive restructuring (negative relation) and minimizing responsibility (positive relation) and dimensions of moral disengagement. Among these associations, cyberaggression emerged as the most relevant statistical connecton.

For cyberaggression based on social stigmas (see Table 4), collinearity analyses were optimal since the values of the variance inflation factor (VIF) were between 1.059 and 2.526 and the tolerance values ranged between 0.360 and 0.944. In addition, the Durbin–Watson statistic revealed a value of 1.698. The regression model revealed that the variables that statistically and positively predict cyberaggression based on social stigmas are cybervictimization and cognitive restructuring, explaining 13.50% of the variance.

## 4. Discussion

The present research was primarily focused on studying the prevalence of involvement roles—cyberaggressor, cybervictim and cyberaggressor/cybervictim—in general cyberbullying and in social stigma-based cyberbullying, as well as the differentiation in this involvement based on gender. The review of the scientific literature revealed a high risk for adolescents becoming involved in cyberbullying based on social stigmas due to ethnic–cultural differences (Calmaestra et al., 2020; Rodríguez-Hidalgo et al., 2018; Graham, 2021; Zych & Llorent, 2023), sexual orientation and/or gender identity (Elipe et al., 2017; Llorent et al., 2016; Garaigordobil & Larrain, 2020) or differences in skills or physical appearance and body image (De Oliveira et al., 2015; Rech et al., 2013; Reulbach et al., 2013). Based on these contributions, a hypothesis was formulated that there would be a prevalence of both social stigma-based cyberbullying and general cyberbullying.

The percentage of those involved as cybervictims based on social stigmas was like the percentage of general cybervictims. However, the percentage of social stigma-based cyberaggressors was lower than that of general cyberaggressors. Regarding the role of cybervictimization/cyberaggression, the percentage of involvement in general cyberbullying is higher than that recorded in cyberbullying based on social stigmas. These conclusions are consistent with previous studies (e.g., Andrade et al., 2021; Calmaestra et al., 2016; Calmaestra et al., 2020), which suggested that involvement in various roles—cyberaggressor, cybervictim, and cyberaggressor/victim—would also be observed in cyberbullying based on social stigmas, as is the case in general cyberbullying. Therefore, the first hypothesis is confirmed. However, the present study also offers findings that further expand on this dimension. In addition, frequency analyses showed that the most common reasons for becoming involved as a cybervictim or cyberaggressor were appearance-based. To date, no prior studies on cyberbullying based on social stigmas have demonstrated this type of evidence. Nevertheless, this conclusion is consistent with the findings of De Oliveira et al. (2015), although their study focused on bullying.

The conclusion of general cyberbullying is that for every ten adolescents, two are recognized as cybervictims; one as a cyberaggressor; and three as both cyberaggressors and cybervictims. Of these cybervictims, almost all say they are targeted due to a discriminatory social stigma. Slightly less than half of the cyberaggressors acknowledged that their actions were based on discriminatory social stigmas. One in four cyberaggressors/cybervictims admitted to being involved in this dynamic due to discriminatory social stigmas. Based on these findings, it can be postulated that general cybervictimization is clearly perceived by adolescents as a form of discrimination based on prevailing social prejudices. However, in contrast, fewer adolescents recognize cyberaggression as being driven by social prejudice. These findings reveal an important insight: adolescents appear to be less likely to identify themselves as cyberaggressors based on social stigmas compared to the extent to which they recognize themselves as cybervictims, in relation to prevailing cultural prejudices. Several factors may contribute to this discrepancy. Some cybervictims, due to belonging to vulnerable identity groups or possessing distinct characteristics, may interpret general acts of cyberaggression as inherently discriminatory. Given the nature of cyberbullying, a significant portion of prejudice-driven attacks may originate from adults, while minors perceive themselves as victims of such stigma-based aggression. On the other hand, some adolescent cyberaggressors may engage in more subtle forms of prejudice-based aggression, and due to the less explicit nature of these behaviors, they may underestimate their discriminatory intent—an intent that their victims are, nevertheless, able to recognize.

Based on the study by Calmaestra et al. (2016) and the report by Andrade et al. (2021), it was expected that boys would surpass girls in involvement in the cyberaggressor role, both in general cyberbullying and in cyberbullying based on social stigma. This second hypothesis was confirmed only for general cyberbullying and not for cyberbullying rooted in social stigmas. In the latter context, girls were more involved than boys in both cybervictimization and cyberaggression related to social stigmas. This gender difference, along with the finding that the most frequently cited reasons for becoming a cybervictim or cyberaggressor were linked to physical and/or esthetic appearance, may be explained by greater social pressure and the stronger imposition of normative standards on girls. As a result, girls may be both more frequently targeted and more frequently engaged as aggressors in appearance-based cyberbullying. Drawing on Social Dominance Theory (Goodboy et al., 2016; Vaillancourt et al., 2003), it is plausible that, among adolescent girls, cyberbullying based on physical and/or esthetic differences is used by aggressors as a means to create or reinforce hierarchical status distinctions with the intent of maintaining or elevating a privileged social position among peers. Conversely, for cybervictims, such appearance-based bullying may be intended to diminish their social status or to facilitate their exclusion from peer groups.

This study also aimed to identify possible links between social stigma-based cyberaggression and social stigma-based cybervictimization, as well as the different dimensions of moral disengagement, moral emotions, and social and emotional competencies, in addition to possible statistical associations between social stigma-based cyberaggression, social stigma-based cybervictimization and the variables described above.

It was concluded that there is a directly proportional relationship between cyberaggression based on social stigmas and the mechanisms of moral disengagement (Bandura, 2002). This finding is consistent with Gini et al. (2014), Zych and Llorent (2019) and Domínguez-Vergara et al. (2023). Additionally, it was revealed that cognitive restructuring—a mechanism of moral disengagement—is the variable that is most closely related to cyberaggression based on social stigmas, along with cybervictimization. This follows the same line as the study of Romera et al. (2021) on general cyberbullying, where cognitive restructuring was the most associated mechanism, specifically enhancing aggression in students. These conclusions support hypothesis 3, as levels of cyberaggression based on social stigmas are positively related to levels of moral disengagement. This seems to indicate that online aggressors make use of cognitive restructuring mechanisms to justify and downplay their aggressive behavior. This may be largely attributed to the depersonalization of the victim enabled by digital devices, which makes it easier for the aggressor to overlook or rationalize the harm caused by their prejudiced actions.

On the other hand, the results of this study disagree with the observations of Perren and Gutzwiller-Helfenfinger (2012) and Oriol Granado (2023), which focused on cyberbullying, as they did not detect a relationship between moral emotions and levels of cybervictimization based on social stigmas. These conclusions do not support hypothesis 4. These findings may be due to differences between the characteristics of the phenomenon when referring to traditional cyberbullying—upon which most previous studies are based—and cyberbullying rooted in social stigmas. This highlights the need for continued research in this area to better understand the specific dynamics of stigma-based aggression. Advancing this line of inquiry is essential, as the prior literature emphasizes the role of socio-emotional competencies in addressing similar forms of violence, as well as the importance of incorporating Social and Emotional Learning (SEL) components to enhance the effectiveness of interventions aimed at reducing stigma-based aggression (Amadori et al., 2023).

The present study revealed positive statistical associations of cybervictimization based on social stigmas: cyberaggression based on social stigmas, cognitive restructuring and the minimization of responsibility. In addition, significant correlations were shown with the responsible decision-making dimension, belonging to the scale of social and emotional competences. The fact that this relationship did not remain significant in the regression analysis could indicate that perhaps this should be studied the opposite direction, that is, whether being a victim of cyberbullying based on social stigmas can lead to worse performance in responsible decision making, something that would be interesting to study further in the future. These findings are consistent with those provided by some previous studies (Domínguez-Vergara et al., 2023; Romera et al., 2016). Therefore, hypothesis 5 was not substantiated. Likewise, both hypothesis 4 and hypothesis 5 could be addressed in future studies by implementing methodological adjustments that would allow for complementary analyses using a larger sample.

## 5. Conclusions

Based on the conclusions of this study, educational inferences can be made to prevent and alleviate stigma-based cyberbullying. Urgent actions are needed to reduce bias-based behavior, which endangers people’s social, personal and emotional development. There are numerous programs to prevent and mitigate cyberbullying, such as Cyberprogram 2.0 (Garaigordobil & Martínez-Valderrey, 2015), which promotes empathy and collaboration, and the ConRed Program (Del Rey et al., 2012), which involves families and schools to raise awareness and prevent bullying and cyberbullying. Additionally, there are programs that incorporate socio-emotional competencies for the prevention of cyberbullying (Martínez-Vilchis et al., 2018). However, these programs must be adapted to specifically address prejudices associated with social stigmas. Effective interventions in the social, educational and political spheres would be necessary to reduce the prevalence of this violent phenomenon of a discriminatory nature. To develop more effective prevention and protection programs, the results of this study can help focus on the most relevant points of intervention. It is proposed that educational programs aimed at preventing and mitigating general cyberbullying and specifically social stigma-based cyberbullying develop learning situations that allow potential cyberaggressors to become aware of their prejudices based on social stigma, as most cybervictims are recognized as being harmed by these prejudices, while cyberaggressors rarely recognize that their attacks are based on social stigma. It is also necessary to include a gender perspective, as boys are more involved in cyberbullying than girls. These programs should primarily focus on developing social and emotional competencies centered on inclusion and diversity, as they promote the inhibition of disruptive and aggressive behaviors, enhancing prosocial behaviors, as evidenced in the quasi-experimental study by Martínez-Vilchis et al. (2018). They should also incorporate educational work to combat the mechanisms of moral disengagement, with particular emphasis on cognitive restructuring and the minimization of responsibility, as they relate both to cybervictimization and cyberaggression based on social stigmas.

In view of the limitations of this study, the data were collected using self-report which provides a lot of information that is sometimes not accessible by other means, but response biases may be present due to the social desirability of the subjects, despite the anonymity of the process. To overcome this possible limitation, future studies should use alternative methodologies, incorporating both self-reports and hetero reports. Additionally, this study employed a cross-sectional design where risk and protective factors were not assessed over time and were abstract from theory, and a relatively small research sample was used. Future research could involve a larger sample through a longitudinal design to better understand predictors, risk and protection factors, and the long-term dynamics of the phenomenon.

This study focused on cyberbullying based on numerous social stigmas, which could be expanded or specifically focus on certain behaviors in future research. It would be interesting to explore the potential feedback loops in this phenomenon, considering the relationship found between cyberaggression and cybervictimization based on stigmas, as well as the connections with the other observed variables. Overall, this study provides data and insights that can inform and promote practices aimed at reducing social stigma-based cyberbullying while also opening new avenues for the further investigation of this phenomenon.

## Figures and Tables

**Table 1:** Analysis of cyberbullying and gender roles.

	Cyberbullying						SSB Cyberbullying					
	Male		Female		Non-Binary		Male		Female		Non-Binary	
	N	%	N	%	N	%	N	%	N	%	N	%
Cybervictimization	69	51.49	64	47.76	1	0.75	53	43.44	68	55.74	1	0.82
Cyberaggression	38	66.67	19	33.33	0	0	8	44.44	10	55.56	0	0
Cybervictimization/Cyberaggression	114	54.03	95	45.02	2	0.95	32	64.00	17	34.00	1	2.00

**Table 2:** Spearman correlations between the roles of cyberbullying, moral disengagement, moral emotions and social and emotional competencies.

	SSB Cybervictimization	SSB Cyberaggression
**SSB cybervictimization**	-	-
**SSB cyberaggression**	0.353 **	-
**Cognitive restructuring**	0.122 **	0.190 **
**Minimizing responsibility**	0.104 *	0.112 **
**Distorting the consequences**	0.072	0.093 *
**Dehumanizing the victim**	0.036	0.132 **
**Guilt**	−0.018	−0.079
**Regret**	0.024	−0.061
**Shame**	0.044	−0.025
**Pride**	0.001	−0.080
**Self-awareness**	−0.072	−0.062
**Self-management and motivation**	−0.052	−0.027
**Social awareness and prosocial behavior**	−0.008	−0.046
**Responsible decision making**	−0.085 *	−0.036

* *p* < 0.05; ** *p* < 0.01. Bold: significant variables.

**Table 3:** Regression model of cybervictimization based on social stigmas.

Adjusted R-Square	Durbin–Watson	Variable	Unstandardized Coefficients		Standardized Coefficients	T	*p*	Collinear Statistics	
			B	Std. Error	Beta			Tolerance	VIF
0.134	1.874	(Constant)	0.192	0.110		1.749	0.081		
		Cyberaggression	0.462	0.059	0.325	7.896	0.000	0.943	1.060
		Cognitive restructuring	0.008	0.004	0.105	1.962	0.050	0.560	1.787
		Minimizing responsibility	0.010	0.005	0.121	2.230	0.026	0.538	1.858
		Distorting the consequences	−0.011	0.009	−0.062	−1.213	0.226	0.608	1.646
		Dehumanizing the victim	−0.010	0.005	−0.106	−1.940	0.053	0.539	1.857
		Guilt	−0.021	0.013	−0.106	−1.595	0.111	0.361	2.766
		Regret	0.040	0.023	0.110	1.731	0.084	0.398	2.513
		Shame	0.021	0.016	0.063	1.267	0.206	0.643	1.555
		Pride	0.003	0.018	0.008	0.162	0.871	0.697	1.435
		Self-awareness	−0.006	0.005	−0.061	−1.175	0.240	0.597	1.674
		Self-management and motivation	−0.001	0.007	−0.005	−0.097	0.923	0.593	1.686
		Social awareness and prosocial behavior	0.004	0.005	0.043	0.749	0.454	0.477	2.098
		Responsible decision making	−0.011	0.007	−0.086	−1.693	0.091	0.617	1.622

Gray background: significant variables.

**Table 4:** Regression model of cyberaggression based on social stigmas.

Adjusted R-Square	Durbin–Watson	Variable	Unstandardized Coefficients		Standardized Coefficients	T	*p*	Collinear Statistics	
			B	Std. Error	Beta			Tolerance	VIF
0.135	1.698	(Constant)	−0.031	0.077		0.900	0.369		
		Cybervictimization	0.228	0.029	0.324	7.896	0.000	0.944	1.059
		Cognitive restructuring	0.007	0.003	0.137	2.584	0.010	0.563	1.777
		Minimizing responsibility	−0.001	0.003	−0.011	−0.195	0.845	0.533	1.875
		Distorting the consequences	−0.001	0.006	−0.005	−0.092	0.927	0.606	1.650
		Dehumanizing the victim	0.004	0.004	0.064	1.176	0.240	0.536	1.865
		Guilt	−0.008	0.009	−0.060	−0.900	0.369	0.360	2.775
		Regret	0.004	0.016	0.017	0.267	0.790	0.396	2.526
		Shame	0.004	0.011	0.018	0.367	0.714	0.641	1.559
		Pride	−0.013	0.013	−0.049	-1.021	0.308	0.698	1.432
		Self-awareness	−0.002	0.004	−0.029	−0.559	0.577	0.596	1.678
		Self-management and motivation	0.003	0.005	0.030	0.579	0.563	0.594	1.684
		Social awareness and prosocial behavior	−0.001	0.004	−0.010	−0.174	0.862	0.476	2.100
		Responsible decision making	0.002	0.005	0.019	0.364	0.716	0.613	1.630

Gray background: significant variables.

## Data Availability

Data are contained within the article.

## Abbreviation

The following abbreviation is used in this manuscript:

SSB	Social stigma-based

## References

[B1-behavsci-15-00646] Amadori A., Sangiuliano-Intra F., Taverna L., Brighi A. (2023). Systematic review of intervention and prevention programs to tackle homophobic bullying at school: A socio-emotional learning skills perspective. International Journal of Prevention.

[B2-behavsci-15-00646] Andersen M. M., Varga S., Folker A. P. (2022). On the definition of stigma. Journal of Evaluation in Clinical Practice.

[B3-behavsci-15-00646] Andrade B., Guadix I., Rial A., Suárez F. (2021). Impacto de la tecnología en la adolescencia. Relaciones, riesgos y oportunidades.

[B4-behavsci-15-00646] Arsenio W. (2014). Moral emotion attributions and aggression. Handbook of moral development.

[B5-behavsci-15-00646] Álamo M., Llorent V. J., Nasaescu E., Zych I. (2019). Validación de la escala de emociones morales en adolescentes. Congreso Internacional de Transferencia de Conocimientos y Sensibilización Social “Islam y Paz a Través de Voces Musulmanas”.

[B6-behavsci-15-00646] Baldry A. C., Farrington D. P., Sorrentino A. (2015). “Am I at risk of cyberbullying”? A narrative review and conceptual framework for research on risk of cyberbullying and cybervictimization: The risk and needs assessment approach. Aggression and Violent Behavior.

[B7-behavsci-15-00646] Bandura A. (2002). Selective moral disengagement in the exercise of moral agency. Journal of Moral Education.

[B8-behavsci-15-00646] Baumeister R. F., Vohs K. D., Nathan DeWall C., Liqing Z. (2007). How emotion shapes behavior: Feedback, anticipation, and reflection, rather than direct causation. Personality and Social Psychology Review.

[B9-behavsci-15-00646] Calmaestra J., Escorial A., García P., Moral Del C., Perazzo C., Ubrich T. (2016). Yo a eso no juego: Bullying y ciberbullying en la infancia *[Archivo PDF]*.

[B10-behavsci-15-00646] Calmaestra J., Rodríguez-Hidalgo A. J., Mero-Delgado O., Solera E. (2020). Cyberbullying in adolescents from Ecuador and Spain: Prevalence and differences in gender, school year and ethnic-cultural background. Sustainability.

[B11-behavsci-15-00646] Caprara G. V., Barbaranelli C., Vicino S., Bandura A. (1995). La Misura del Disimpegno Morale (The assessment of moral disengagement). Rassegna Di Psicología 13.

[B12-behavsci-15-00646] Cuadrado I., Fernández I., Martín-Mora Parra G. (2019). ¿Pueden las víctimas de bullying convertirse en agresores del ciberespacio? Estudio en población adolescente. European Journal of Investigation in Health, Psychology and Education.

[B13-behavsci-15-00646] De Oliveira W. A., Silva M. A. I., de Mello F. C. M., Porto D. L., Yoshinaga A. C. M., Malta D. C. (2015). The causes of bullying: Results from the National Survey of School Health (PeNSE). Revista Latino-Americana de Enfermagem.

[B14-behavsci-15-00646] Del Rey R., Casas J. A., Ortega R. (2012). El programa ConRed, una práctica basada en la evidencia. Comunicar.

[B15-behavsci-15-00646] Del Rey R., Casas J. A., Ortega-Ruiz R., Schultze-Krumbholz A., Scheithauer H., Smith P., Thompson F., Barkoukis V., Tsorbatzoudis H., Brighi A., Guarini A., Pyżalski J., Plichta P. (2015). Structural validation and cross-cultural robustness of the European cyberbullying intervention project questionnaire. Computers in Human Behavior.

[B16-behavsci-15-00646] Domínguez-Vergara J., Santa-Cruz-Espinoza H., Chávez-Ventura G., Ybañez-Carranza J. (2023). La desconexión moral como mediadora entre la agresividad y el ciberacoso en escolares. Revista Internacional de Sociología de la Educación.

[B17-behavsci-15-00646] Eden S., Heiman T., Olenik-Shemesh D. (2016). Bully versus victim on the internet: The correlation with emotional-social characteristics. Education and Information Technologies.

[B18-behavsci-15-00646] Elipe P., de la Oliva Muñoz M., Del Rey R. (2017). Homophobic bullying and cyberbullying: Study of a silenced problem. Journal of Homosexuality.

[B19-behavsci-15-00646] Fundación ANAR, Fundación Mutua Madrileña (2022). La opinión de los estudiantes: IV informe de prevención del acoso escolar en centros educativos.

[B20-behavsci-15-00646] Garaigordobil M., Larrain E. (2020). Bullying and cyberbullying in LGBT adolescents: Prevalence and effects on mental health. Comunicar.

[B21-behavsci-15-00646] Garaigordobil M., Martínez-Valderrey V. (2015). Effects of Cyberprogram 2.0 on “face-to-face” bullying, cyberbullying and empathy. Psicothema.

[B22-behavsci-15-00646] Gini G., Pozzoli T., Hymel S. (2014). Moral disengagement among children and youth: A meta-analytic review of links to aggressive behavior. Aggressive Behavior.

[B23-behavsci-15-00646] Goodboy A. K., Martin M. M., Rittenour C. E. (2016). Bullying as a display of social dominance orientation. Communication Research Reports.

[B24-behavsci-15-00646] Gómez-Urrutia V., Jiménez Figueroa A. (2022). Identidad en la era digital: Construcción de perfiles en redes sociales en adolescentes chilenos/as. Convergencia.

[B25-behavsci-15-00646] Graham S. (2021). Exploration of identity-based bullying by race/ethnicity and other marginalized identities among adolescents. JAMA Network Open.

[B26-behavsci-15-00646] Instituto Nacional de Estadística [INE] (2022). Encuesta sobre equipamiento y uso de tecnologías de información y comunicación (TIC) en los hogares. Año 2022.

[B27-behavsci-15-00646] Instituto Nacional de Estadística [INE] (2024). Encuesta sobre equipamiento y uso de tecnologías de información y comunicación (TIC) en los hogares. Año 2024.

[B28-behavsci-15-00646] Kowalski R. M., Limber S. P., McCord A. (2019). A developmental approach to cyberbullying: Prevalence and protective factors. Aggression and Violent Behavior.

[B29-behavsci-15-00646] Llorent V. J., Diaz-Chaves A., Zych I., Twardowska-Staszek E., Marín-López I. C. (2021). Bullying and cyberbullying in Spain and Poland, and their relation to social, emotional and moral competencies. Salud Mental Escolar.

[B30-behavsci-15-00646] Llorent V. J., Ortega-Ruiz R., Zych I. (2016). Bullying and cyberbullying in minorities: Are they more vulnerable than the majority group?. Frontiers in Psychology.

[B31-behavsci-15-00646] Marinoni C., Rizzo M., Zanetti M. A. (2024a). Fake profiles and time spent online during the COVID-19 pandemic: A real risk for cyberbullying?. Current Psychology.

[B32-behavsci-15-00646] Marinoni C., Rizzo M., Zanetti M. A. (2024b). Social media, online gaming, and cyberbullying during the COVID-19 pandemic: The mediation effect of time spent online. Adolescents.

[B33-behavsci-15-00646] Martínez-Vilchis R., Morales Reynoso T., Pozas Rivera J. (2018). Efectos de un programa de competencias emocionales en la prevención de cyberbullying en bachillerato. Pensamiento Psicológico.

[B34-behavsci-15-00646] Montero I., León O. G. (2007). A guide for naming research studies in psychology. International Journal of clinical and Health psychology.

[B35-behavsci-15-00646] Oriol Granado X. (2023). Prevención de las distintas formas de ciberacoso y acoso tradicional entre iguales: El papel de las emociones morales. Infancia y Adolescencia.

[B36-behavsci-15-00646] Ortega-Ruiz R. (2018). El bullying y el ciberbullying como expresión de una agresividad injustificada e inmoral. Anales de la Fundación Canis Majoris.

[B37-behavsci-15-00646] Ortega-Ruiz R., Del Rey R., Casas J. A. (2016). Evaluar el bullying y el cyberbullying validación española del EBIP-Q y del ECIP-Q. Psicología Educativa.

[B38-behavsci-15-00646] Ortega-Ruiz R., Sánchez V., Menesini E. (2002). Violencia entre iguales y desconexión moral: Un análisis transcultural. Psicothema.

[B39-behavsci-15-00646] Perren S., Gutzwiller-Helfenfinger E. (2012). Cyberbullying and traditional bullying in adolescence: Differential roles of moral disengagement, moral emotions, and moral values. European Journal of Developmental Psychology.

[B40-behavsci-15-00646] Rech R. R., Halpern R., Tedesco A., Santos D. F. (2013). Prevalência e características de vítimas e agressores de bullying. Jornal de Pediatria.

[B41-behavsci-15-00646] Reulbach U., Ladewig E. L., Nixon E., O’Moore M., Williams J., O’Dowd T. (2013). Weight, body image and bullying in 9-year-old children: Weight and bullying. Journal of Paediatrics and Child Health.

[B42-behavsci-15-00646] Rodríguez-Hidalgo A. J., Solera E., Calmaestra J. (2018). Psychological predictors of cyberbullying according to ethnic-cultural origin in adolescents: A national study in Spain. Journal of Cross-Cultural Psychology.

[B43-behavsci-15-00646] Romera E. M., Casas J. A., Gómez-Ortiz O., Ortega-Ruiz R. (2019a). Moral domain as a risk and protective factor against bullying. An integrating perspective review on the complexity of morality. Aggression and Violent Behavior.

[B44-behavsci-15-00646] Romera E. M., García-Fernández C. M., Ortega-Ruiz R. (2016). Cyberbullying: Competencia social, motivación y relaciones entre iguales. Comunicar.

[B45-behavsci-15-00646] Romera E. M., Herrera-López M., Ortega-Ruiz R., Camacho A. (2023). The moral disengagement scale-24: Factorial structure and cross-cultural comparison in Spanish and Colombian adolescents. Psychology of Violence.

[B46-behavsci-15-00646] Romera E. M., Ortega-Ruiz R., Rodríguez-Barbero S., Falla D. (2019b). How do you think the victims of bullying feel? A study of moral emotions in primary school. Frontiers in Psychology.

[B47-behavsci-15-00646] Romera E. M., Ortega-Ruiz R., Runions K., Falla D. (2021). Moral disengagement strategies in online and offline bullying. Intervención Psicosocial.

[B48-behavsci-15-00646] Ruiz-Olabuénaga J. I. (2012). Teoría y práctica de la investigación cualitativa. Ph.D. thesis.

[B49-behavsci-15-00646] Smith P., Mahdavi J., Carvalho M., Fisher S., Russell S., Tippett N. (2008). Cyberbullying: Its nature and impact in secondary school pupils. Journal of Child Psychology and Psychiatry.

[B50-behavsci-15-00646] Solsol-Urquía S. (2022). Ciberbullying y competencias socioemocionales en estudiantes de secundaria de una institución educativa. Ph.D. thesis.

[B51-behavsci-15-00646] Vaillancourt T., Hymel S., McDougall P. (2003). Bullying is power: Implica-tions for school-based intervention strategies. Journal of Applied School Psychology.

[B52-behavsci-15-00646] Wright M. F., Wachs S. (2021). Does empathy and toxic online disinhibition moderate the longitudinal association between witnessing and perpetrating homophobic cyberbullying?. International Journal of Bullying Prevention.

[B53-behavsci-15-00646] Zych I., Llorent V. J. (2019). Afective empathy and moral disengagement related to late adolescent bullying perpetration. Ethics and Behavior.

[B54-behavsci-15-00646] Zych I., Llorent V. J. (2023). Bias-based cyberbullying in Spanish adolescents and its relation to social and emotional competencies and technology abuse. The Journal of Early Adolescence.

[B55-behavsci-15-00646] Zych I., Ortega-Ruiz R., Muñoz-Morales R., Llorent V. J. (2018). Dimensions and psychometric properties of the social and emotional competencies questionnaire (SEC-Q) in youth and adolescents. Revista Latinoamericana de Psicología.

